# Postfire responses of the woody flora of Central Chile: Insights from a germination experiment

**DOI:** 10.1371/journal.pone.0180661

**Published:** 2017-07-12

**Authors:** Susana Gómez-González, Susana Paula, Lohengrin A. Cavieres, Juli G. Pausas

**Affiliations:** 1 Departamento de Biología-IVAGRO, Universidad de Cádiz, Puerto Real, España; 2 Centre for Science and Resilience Research [(CR)2], Universidad de Chile, Santiago, Chile; 3 Instituto de Ciencias Ambientales y Evolutivas, Universidad Austral de Chile, Valdivia, Chile; 4 ECOBIOSIS, Departamento de Botánica, Facultad de Ciencias Naturales y Oceanográficas, Universidad de Concepción, Concepción, Chile; 5 Instituto de Ecología y Biodiversidad (IEB), Santiago, Chile; 6 Centro de Investigación sobre Desertificación (CIDE-CSIC), Valencia, España; Estacion Experimental de Zonas Aridas, SPAIN

## Abstract

Fire is a selective agent shaping plant traits and community assembly in fire-prone ecosystems. However, in ecosystems with no fire history, it can be a cause of land degradation when it is suddenly introduced by humans, as plant species may not be able to respond to such novel disturbance. Unlike other Mediterranean-type ecosystems (MTE) of the world, natural fires have not been frequent during the Quaternary in the matorral of Central Chile, and thus, plant adaptive responses are expected to be uncommon. We evaluated the effect of heat shock on seed survival and germination of 21 native woody plants of the Chilean matorral and compiled information on smoke-stimulation and resprouting, to evaluate the importance of fire-adaptive responses in the context of the other MTE. We found that in the Chilean woody flora negative seed responses to fire cues were more frequent than positive responses. Although resprouting is a relatively widespread trait, fire-stimulated germination is not as common in the Chilean matorral as in other MTE. The seeds of seven endemic species were strongly damaged by fire cues and this should be considered in post-fire restoration planning. However, our results also showed that many species were resistant to elevated doses of heat shock and in some, germination was even stimulated. Thus, future research should focus on the evolutionary causes of these responses. These findings could help to develop strategies for fire management in the Chilean matorral. In addition, they will improve our understanding of the evolutionary forces that shaped this plant community and to better frame this region among the other MTE worldwide.

## Introduction

Fire is a disturbance that, in many ecosystems, shapes plant evolution and community assemblage at different spatial and temporal scales [1–7]. In regions where wildfires have been historically frequent (e.g., most Mediterranean-type ecosystems and tropical savannas) plants have acquired traits that allow them to quickly recover after fire [8–10]. By contrast, in ecosystems where fire has been recently introduced by human activities, plant communities are more vulnerable to postfire degradation and invasion by introduced species [11–13].

Mediterranean-type ecosystems (MTE) from California, South Africa, SW Australia and the Mediterranean Basin have been subjected to natural (lightning-ignited) fire regimes since (at least) the Pliocene [14–15, 10]. In these ecosystems, many plant species are able to persist after fire by having thick bark [7, 16], resprouting from dormant buds [17–18], and/or recruiting new individuals from the seed bank (located either in the soil or in the canopy; [19–22]) or from new seeds produced after fire-stimulated flowering [23]. High resource availability, weak competition, and low seed predation after fire, increase the success of seedling establishment. Thus, the seeds of many species from MTE did not only evolve to resist the high temperatures of fires, but acquired mechanisms to increase their postfire germination and recruitment (e.g., heat and smoke-stimulated germination), in order to rapidly replenish the seed bank before the next fire event [21, 24–25]. According to Pausas & Keeley [26], changes in fire regime (at an evolutionary timescale) would determine variations in the relative importance of resprouting and post-fire seedling recruitment due to ecological trade-offs between these fire-related traits. Specifically, they proposed that species with fire-stimulated germination (seeders) should have evolved in MTE as a response to recurrent high-intensity fires, concomitant to the loss of the resprouting ability.

Mediterranean-type ecosystems of Central Chile (the matorral) stand out among the other MTE because their current fire regime is recent (Holocene) and mainly driven by human activities [27–28]. Although natural fires were relatively common through the Miocene [29], the Andean uplift in the late-Miocene formed an effective barrier to westward storms. This barrier, together with the decrease in the air temperature due to the cold Humboldt oceanic current, reduced the frequency of lightning, and thus the occurrence of lightning-initiated fires [10, 30–31]. The extent to which this complex fire history has affected the fire adaptations of plants remains to be explored in detail. The existence of lignotubers in native woody species (a specialized postfire resprouting organ; [6, 18, 32]) and the low recruitment of seedlings observed in burned areas [33], suggest that a reduction in fire frequency during the Holocene (compared to Early-Middle Miocene [29]) might have caused the loss of seeder syndromes [10].

Here, we evaluate the germination response of common woody plants native to the Chilean matorral, following fire-related cues of heat and smoke. Studies on this topic are few (e.g., [34–36]) and focus on soil seed banks dominated by exotic herbaceous species [37–41]. There is evidence that the native seed bank can be destroyed by high-severity fires, but can survive fire of low severity [33–34, 37]. Gómez-González et al. [35] evaluated the effect of smoke on the seed germination of 18 native woody plants of the Chilean matorral, concluding that although smoke inhibited the germination in typical species of mature (undisturbed) matorral communities, it stimulated the germination of some pioneer species. Nevertheless, there are no conclusive studies assessing the role of heat shock on seed germination of native matorral species (see [34] for some descriptive results on seven species). This is a limitation given that heat-stimulated germination is a common and well-studied trait in other MTE [21, 25]. Therefore, for a comprehensive assessment of the role of fire on the germination of Chilean native woody species, we need information about the effects of high temperatures on seed viability and germinability.

Based on the Pausas & Keeley [26] model and the fire history of the region (a significant reduction of high-intensity fires during the Quaternary compared to Early-Middle Miocene), we hypothesized that negative seed responses to fire cues will be more frequent than favorable responses among woody species from the Chilean matorral. Therefore, fire-stimulated germination in the matorral should be less relevant than in other MTE. To test this hypothesis, we evaluated the effect of different heat doses on the seed survival and germination of 21 common native woody plants of the Chilean matorral. We also considered the available information on smoke-stimulated germination and resprouting capacity of the matorral species in order to make an overall evaluation of their postfire response, and thus to frame this ecosystem among the other MTE.

## Materials and methods

### Seed sampling

Seeds of 21 native woody species (12 shrubs and 9 trees; including 14 endemics; [42]) were collected at different localities along the Coastal Range and the Andes foothills in Central Chile (32–38°S; Metropolitan, Valparaíso and Bío-Bío regions) (Table 1 [33–34, 37, 43–47]; S1 Table). The selected species include dominant species from typical lowland sclerophyllous matorral and montane sclerophyllous woodlands, widely distributed in Central Chile [48]. Each species was collected in one locality, except in the case of *Colliguaja odorifera*, in which seeds from three locations were pooled due to low seed production (S1 Table). Plant individuals were randomly selected in variable number depending on the population density of each species. According to the literature, at least 86% of the studied species can resprout after fire, and post-fire recruitment has been observed in 48%, either in field studies or after experimental litter burning on soil seedbanks (Table 1 [33–34, 37, 43–47]).

**Table 1:** List of studied species. Most of them are dominant species in the Chilean matorral.

Species	Family	Growth form	Post-fire recruitment	Post-fire resprouting
***Acacia caven***	Fabaceae	Tree	yes-low [37]	yes[44–45]
***Azara petiolaris* (E)**	Salicaceae	Shrub	—	yes [33]
***Baccharis linearis***	Asteraceae	Shrub	yes-low [33]	yes [45]
***Buddleja globosa***	Asteraceae	Shrub	—	no [45]
***Cestrum parqui***	Solanaceae	Shrub	—	yes [45]
***Colliguaja integerrima***	Euphorbiaceae	Shrub	—	—
***Colliguaja odorifera* (E)**	Euphorbiaceae	Shrub	yes[34]	yes [43–46]
***Cryptocarya alba* (E)**	Lauraceae	Tree	yes-low [33]	yes[44–45,47]
***Kageneckia angustifolia* (E)**	Asteraceae	Tree	—	—
***Kageneckia oblonga* (E)**	Asteraceae	Tree	—	yes [45]
***Lithraea caustica* (E)**	Anacardiaceae	Tree	yes-low [33,43]	yes [33,43–47]
***Maytenus boaria***	Celastraceae	Tree	yes-low [33]	yes[45]
***Muehlenbekia hastulata***	Polygonaceae	Woody vine	yes[33–34]	yes[33,45]
***Peumus boldus* (E)**	Monimiaceae	Tree	—	yes[33,45]
***Podanthus mitiqui* (E)**	Asteraceae	Shrub	yes-low [33,43]	variable[43,45]
***Otholobium glandulosum* (E)**	Fabaceae	Shrub	—	yes[45]
***Quillaja saponaria* (E)**	Rosaceae	Tree	yes[34]	yes [33,43–45]
***Retanilla ephedra* (E)**	Rhamnaceae	Shrub	—	yes[45]
***Retanilla trinervia* (E)**	Rhamnaceae	Shrub	yes [33–34,43]	yes-high [33,43,45]
***Senna candolleana* (E)**	Fabaceae	Shrub	—	yes[45]
***Sophora macrocarpa* (E)**	Fabaceae	Tree	—	yes[33,45]

The family, growth form, and the ability of post-fire recruitment and/or resprouting are also included. (E) = endemic to Chile. (—) Not evaluated or questionable information. (low) observation after low severity fires; (high) observation after high severity fires. Nomenclature and endemic status follow Zuloaga et al. [42].

Mature seeds and fruits of all species were collected between late summer and late autumn (depending on the species phenology), and stored in paper bags at room temperature in the laboratory until the beginning of the experiments. Seed collection and experimental assays were performed during 2013–2014, except for the species *Muehlenbeckia hastulata*, *Lithraea caustica* and *Baccharis linearis*, which were collected in 2008 and experimentally tested in 2009 (S1 Table). The mean storage time of the seeds was 9.7 ± 4 months (n = 21 species, S1 Table).

The National Forest Service (CONAF, Chile) gave us permission to take seed samples at Río Clarillo National Reserve (Pirque), La Campana National Park (Olmué) and Yerba Loca Sanctuary of Nature (Lo Barnechea). The Association Parque Cordillera gave us permission to take seed samples at Aguas de Ramón Natural Park (La Reina). The remaining localities (S1 Table) were on private land and permission was granted by the land-owners. This study did not involve endangered or protected species.

### Experimental assay

Seeds of the 21 studied species were subjected to three treatments: 100°C during 5 min, 120°C during 5 min, and untreated seeds (control). These heat doses were selected based on the top soil temperature reached after burning matorral woody litter [37], and on previous literature from other MTE (e.g., [25]). Treatments were performed in a drying oven (Heraeus Function Line T6, ThermoScientific^TM^, USA), and setting the temperature at 2°C above the target temperature in order to diminish the cooling effect when opening the door.

We used a randomized block design, using from 25 to 50 seeds per block and four or six blocks per treatment (S1 Table); the number of seeds per block and the number of blocks varied among species due to differences in seed availability. The seeds of each block were placed into an aluminum tray, and the trays of different species randomly placed in the center of the oven to minimize the effect of potential temperature variation within it. Only one block per species was introduced in the oven at the same time, in order to avoid pseudo-replication. After the treatment application, the seeds of each tray were transferred to a Petri dish with absorbent paper, then watered with distilled water and placed into a germination chamber under controlled conditions (12 h light at 20°C and 12 h dark at 10°C; simulating autumn conditions, when germination of the studied species occurs). All Petri dishes were watered and checked for seedling emergence every two days, during 36 days. After that, we determined the viability of non-germinated seeds using the tetrazolium test (TTC 1% in phosphate buffer, pH 7.3, and 24 h in darkness). We considered surviving seeds those germinated during the monitoring period plus non-germinated seeds identified as viable by the TTC test.

In the case of *Cryptocarya alba*, the pericarp of the seeds was manually removed before the experiments since it is known that it has inhibitory effects on seed germination ([49]; see also S1 Supporting Information). The species evaluated in 2009 (*M*. *hastulata*, *L*. *caustica* and *B*. *linearis*) were only subjected to the treatment of 100°C (5 min) and seed viability was not tested after the germination assay; they belonged to a different data set from a previous study (S. Gómez-González, unpublished data), and were included here because they are very frequent species in the matorral [48], and their seedlings have been observed after fire [33–34].

### Statistical analyses

For each studied species, we performed Generalized Linear Mixed Models (GLMM) to assess the effect of each heat treatment (100°C and 120°C) on the probability of seed germination and seed survival. We included the Petri dish as a random factor (block), and used the logit link function (binomial error distribution). The effect of each heat treatment was tested separately (only compared to control) because: 1) not all species were tested for the effect of 120°C treatment; 2) in many cases there were no germination under 120°C treatment, generating problems of model convergence. The effect of 100°C on seed germination was addressed in 21 species, while the effect of 120°C on germination and the effect of 100°C and 120°C on seed survival was assessed in 18 species (i.e., all except *L*. *caustica*, *M*. *hastulata* and *B*. *linearis*; see above). We performed Generalized Linear Models (Glz; binomial data distribution) to test whether seed storage time had some influence on the seed responses to the experimental treatments across species, without finding any significant effect (S2 Table).

## Results

Seven out of the 21 species evaluated (33%) showed a positive seed response (survival or germination) to some level of heat-shock, while 10 species (48%) had a negative response and four species (19%) were unaffected (Table 2 [35, 50]).

**Table 2:** Effect of heat shock treatments (100°C and 120°C) on the percentage of seed germination and seed survival of 21 common woody species from the Chilean matorral.

Species	Germination (%)			Survival (%)			Seed response to heat	Germinative response to smoke	Estimated fire response
	Control	100°C	120°C	Control	100°C	120°C			
***Acacia caven***	5.6 (2.4)	3.6 (2.2)	2.1 (0.8)	94.7 (5.2)	95.8 (3.6)	97.9 (1.7)	0	G[35]	Stimulated
***Azara petiolaris* (E)**	59.1 (19.3)	0^nc^ (0)	0^nc^ (0)	58.3 (19.3)	0^nc^ (0)	0^nc^ (0)	-	?	Inhibited
***Baccharis linearis***	78.9 (3.9)	80.6 (3.4)	NA	NA	NA	NA	0	0[50]	Unaffected
***Buddleja globosa***	8.7 (2.6)	1.9^*^ (1)	0^nc^ (0)	13.8 (2.1)	1.9^***^ (1)	0^nc^ (0)	-	0[50]	Inhibited
***Cestrum parqui***	98.5 (1.8)	0^nc^ (0)	0^nc^ (0)	98.5 (1.8)	9.5^***^ (3.7)	15^***^ (9.7)	-	?	Inhibited
***Colliguaja integerrima***	65.0 (2.1)	14.0^***^ (4)	0^nc^ (0)	76 (8.3)	67 (1.3)	71 (7.9)	-	0[50]	Inhibited
***Colliguaja odorifera* (E)**	31.9 (5.6)	38.1 (1.7)	4.4^***^ (1.7)	67.5 (3.4)	73.1 (3.2)	51.9^*^ (6.1)	-	NG[35]	Unaffected-Low/ Inhibited-High
***Cryptocarya alba* (E)**	82.2 (1.2)	76.8 (1)	80.0 (2.2)	82.2 (1.2)	76.8 (2.2)	80 (1)	0	-[35]	Inhibited
***Kageneckia angustifolia* (E)**	90.4 (2.7)	91.5 (3.9)	7.5^***^ (5.2)	90.4 (2.7)	91.5 (3.9)	13^***^ (5.2)	-	-[35]	Inhibited
***Kageneckia oblonga* (E)**	95.5 (1.5)	95.5 (2.4)	41.0^***^ (7.8)	95.5 (1.5)	95.5 (2.4)	53.5^***^ (7.7)	-	-[35]	Inhibited
***Lithraea caustica* (E)**	4.4 (1.4)	25.6^***^ (3.7)	NA	NA	NA	NA	G	Variable[35,50]	Stimulated-Low
***Maytenus boaria***	6.2 (3.3)	1.2 (1)	0^nc^ (0)	66.3 (6.2)	45 (11.5)	0^nc^ (0)	-	-[35]	Inhibited
***Muehlenbekia hastulata***	0.6 (0.4)	5.6^*^ (0.8)	NA	NA	NA	NA	G	G[50]	Stimulated-Low
***Peumus boldus* (E)**	0 (0)	0 (0)	0 (0)	50 (16.3)	72.5^*^ (12.6)	92.5^***^ (5)	S	NG[50]	Unaffected
***Podanthus mitiqui* (E)**	61.0 (6.1)	0^nc^ (0)	0^nc^ (0)	61 (6.1)	12.5^*^ (9.5)	7.5^**^ (7.5)	-	0[50]	Inhibited
***Otholobium glandulosum* (E)**	87.5 (1.7)	94.0^*^ (2.4)	14.7^***^ (3.3)	99.5 (0.5)	98.5 (1.5)	14.7^***^ (3.3)	G	?	Stimulated-Low/ Inhibited-High
***Quillaja saponaria* (E)**	15.5 (4.1)	11.0 (3.7)	0^nc^ (0)	45 (4.9)	51 (10.2)	62 (12.5)	-	0[50]	Inhibited
***Retanilla ephedra* (E)**	5.1 (0.8)	7.5 (0.6)	13.7 (4.2)	78.4 (1.9)	80 (1.6)	82.5 (2.6)	0	?	Unaffected
***Retanilla trinervia* (E)**	4.0 (1.4)	7.0 (1)	20.0^***^ (2.2)	74 (8.6)	65 (9.1)	74 (4.8)	G	NG[50]	Stimulated-High
***Senna candolleana* (E)**	3.5 (0.5)	9.0^*^ (2.4)	2.0 (1.2)	69 (2.4)	98^***^ (2)	100^***^ (0)	GS	?	Stimulated-High
***Sophora macrocarpa* (E)**	17.5 (2.8)	22.5 (4)	35.8^*^ (3.9)	69.2 (6.2)	97.5^***^ (1)	85 (4.4)	GS	?	Stimulated-High

The estimated fire response is also shown, based on the available information on seed responses to fire cues (heat and smoke).The mean value per block is shown (see S1 Table for the number of seeds and blocks of each species). SD values are between parentheses. Asterisks indicate significant differences between control and each heat treatment

(*) P < 0.05

(**) P < 0.01

(***) P < 0.001

(nc) = not converged model due to zero values just under heat shock treatment (a negative effect was assumed; GLMM). (E) = endemic to Chile. NA = Not addressed. Seed responses are codified by letters and signs: (G) Stimulated germination; (S) Increased survival. (-) Inhibition; (0) unaffected; (NG) no germination; (?) Unknown. Low = Response expected after low intensity fires; High = Response expected after high intensity fires. Nomenclature and endemic status follow Zuloaga et al. [42].

Considering seed germination after 100°C, four out of 21 species (19%) were stimulated, five species (24%) were inhibited, and 12 species (57%) were not affected. After 120°C, germination of two out of the 18 species (11%) evaluated were stimulated, 11 species (61%) were inhibited and five (28%) unaffected (Table 2 [35, 50]; S3 Table). Regarding seed survival after 100°C, there were positive effects in three species (17%), negative effects in four species (22%), and 11 species (61%) were unaffected. In the case of 120°C treatment, seed survival increased in two species (11%) and decreased in nine species (50%); seven species (39%) were unaffected (Table 2 [35, 50]; S3 Table).

In *Retanilla trinervia*, *Senna candolleana*, *Maytenus boaria*, *Retanilla ephedra*, *Acacia caven*, and *Peumus boldus*, control samples had low percentages of seed germination (<10%) but high seed viability (>50%), indicating that these species had some level of dormancy (Table 2 [35, 50]).

## Discussion

Our results showed that favorable seed responses to heat shock were less frequent among the native matorral species than negative responses (33% vs. 48% of the evaluated species, respectively). However, it is noticeable that 52% of the assessed species were resistant to heat-shock treatments. Indeed, we found evidence of heat-stimulated germination in native species that are common in disturbed matorral communities (e.g., *L*. *caustica*, *M*. *hastulata*, *R*. *trinervia*, *S*. *candolleana* and *S*. *macrocarpa*). Consistently, Gómez-González et al. [35] reported smoke-stimulated germination in some pioneer woody species of the matorral, such as *A*. *caven* and *Baccharis vernalis*. In addition, studies focused on soil seed banks have showed that low-severity fires favored the seedling emergence of many native herbs (e.g., *Bromus berteroanus*, *Dichondra sericea*, *Helenium aromaticum*, *Clarkia tenella*, *Gamochaeta spiciformis*, *Plagiobothrys fulvus* and *Loasa tricolor*; [37–38]), and as consequence, the species richness of this group of plants can increase in burned areas [39]. This evidence rejects the hypothesis of absence of fire-stimulated germination among matorral species proposed in previous studies (e.g., [32, 51]).

Notwithstanding, germination stimulated by fire cues seems to be proportionally less represented in the woody flora of Central Chile compared to the other MTE. Considering our results in addition to other published evidence [34–35], germination is stimulated by heat and/or smoke in only eight out of the 29 species evaluated so far (27.6%) (S4 Table [34–35]). By contrast, fire-stimulated germination has been reported in around 50% of the species evaluated in different studies from the MTE of California, Australia and South Africa [24, 52–55], and in some areas of the Mediterranean Basin, this frequency might be even higher [25]. Additionally, none of the species we studied exclusively rely on fire for their recruitment, contrarily to many species from other MTE [21]. These results, together with the fact that most woody species in the matorral are resprouters [32], strongly suggest that fire has not played a relevant role in shaping plant traits in this ecosystem; in other MTE recurrent fires have selected against resprouting in many taxa, favoring the acquisition of traits enhancing post-fire recruitment [26, 56–57]. In fact, all the studied species with heat-stimulated germination are resprouters and belong to hard-seeded families (e.g., Fabaceae, Rhamnaceae and Anacardiaceae; [58]), suggesting a possible phylogenetic effect determining the presence of seed traits that are beneficial under current fire conditions but that were not evolutionary shaped by fire in the recent history. On the other hand, these species are dispersed by animals (mainly birds and ungulates), where the chemical scarification after gut digestion can have similar effects than heat-shock in breaking the physical dormancy of their seeds [59]. Therefore, the favorable responses to heat-shock found here could be also the result of exaptive processes (*sensu* Gould & Vrba [60]). Nonetheless, worth to mention is the case of *A*. *caven* where simulated cattle ingestion did not influence seed germination [61], nor heat-shock treatments (this study), but plant-derived smoke can break its seed dormancy [35]. Therefore, the possibility of fire adaptations among native matorral species cannot be discarded considering the recent discovery of Miocene fires [29] and the selection of heritable seed traits by anthropogenic fires in a native annual [4–5].

Differences in the criteria used on the selection of species might also explain the variation in the proportion of fire-stimulated germination across MTE [62]. For instance, Moreira et al. [25] reported smoke- and/or heat-stimulated germination in 77% out of 30 Mediterranean species from Spain. They selected the species according to field observations of successful post-fire recruitment, whilst in this study we wanted to analyze the seed responses to heat shock in some of the most frequent woody species from the matorral [48] as an insight for a better understanding of the post-fire regeneration in this ecosystem. In this sense, all the studied species for which post-fire recruitment were previously reported (mainly after low-severity fires, Table 1 [33–34, 37, 43–47]), showed seed tolerance to (at least) 100°C heat shock and/or smoke-stimulated germination (Table 2 [35, 50]). Thus, available observations of post-fire recruitment for the woody species of the matorral are consistent with their responses to experimental fire cues.

Interestingly, seed responses were also consistent among experimental treatments (different doses of heat and smoke); in general, species inhibited by 100°C heat shock where also inhibited by 120°C in a similar or stronger way and species with heat-stimulated germination were also stimulated or not affected by smoke (and vice versa). As consequence, we could make a simple classification of the estimated fire-responses of the studied species (Table 2 [35, 50]). However these predictions need to be tested in the field in order to improve the (scarce) current information on ecological patterns after fire in the Chilean matorral.

Some of the species evaluated here maintained a high proportion of viable seeds despite their low germination in all treatments (e.g., *A*. *caven*, *M*. *boaria*, *Q*. *saponaria*, *C*. *odorifera*, *P*. *boldus*, *R*. *ephedra)*. Even those species stimulated by heat-shock (e.g., *R*. *trinervia*, *S*. *candolleana*, *S*. *macrocarpa*) maintained a high proportion of viable seeds that did not germinate. These species may have different mechanisms of seed dormancy (physical or physiological; [59]) allowing them to form a persistent seed bank that is resistant to fire, but cued by other agents (e.g., light, nutrients, endozoochory). Therefore, a staggered germination could occur in subsequent seasons after fire. Alternatively, higher doses of heat shock or the combination of heat with smoke (or ashes) might trigger their germination [21].

Seed survival surprisingly increased after the heat shock treatment in three species: *P*. *boldus*, *S*. *candolleana* and *S*. *macrocarpa*. In these cases, we observed that heated samples were less infected by fungi than the control samples (S1 Fig). This might indicate an indirect positive effect of fire on seed germination through heat-induced fungi mortality [63–64], although these effects might only be relevant in the short-term or under laboratory conditions. In any case, the evolution of plant-soil interactions in response to fire is a topic that deserves more attention.

Our results have implications for management and conservation of this threatened and highly diverse ecosystem [65]. Sixty seven percent (14) of our studied species are endemics, and seven of them were inhibited by heat or smoke. The negative effect of burning would be especially high in the case of severe crown fires, since the highest heat doses applied in our experiments (120°C, 5 min) reduced the seed germination in 48% of the evaluated species, and the native seed bank can be completely destroyed when soil temperature surpasses 130°C [33–35]. Therefore, recurrent high-severity fires could not only deplete their seedbanks but also their resprouting ability, having strong detrimental effects on their population dynamics. In this sense, active restoration techniques after fire including manual seeding and planting of endangered endemic plants would be desirable. Finally, considering that intense fires are particularly dangerous for the maintenance of native species seedbanks, management policies could be designed to reduce the additional fuel load imposed by some flammable exotic species that frequently invade natural areas (e.g., *Pinus radiata*, *P*. *contorta*, *Acacia dealbata*, *Teline mospesulana*, *Ulex europaeus*), and increase fire proneness [66–69].

## Conclusions

Negative seed responses to fire cues are more frequent than positive responses in common native woody plants from Central Chile, suggesting that the matorral would not be a typical “fire-adapted” plant community. Chilean plants have experienced abrupt changes in fire regime throughout their evolutionary history, compared with other MTE, and this can be traced in their different responses to fire. While in most MTE fire-stimulated germination is a prominent and widespread trait, in the Chilean matorral flora this is less relevant and confined to pioneering species. Notwithstanding, the seeds of many species are resistant to elevated doses of heat (> 50% seed survival after 120°C heat-shock) and some exhibit heat-stimulated germination. Future research should be dedicated to address the evolutionary causes of these adaptive responses to fire. In addition, our results have direct management implications, since there are several endemic species that are expected to be damaged by recurrent severe fires; they should have priority for being restored in burned areas. This study contributes to our understanding of the fire ecology and management of the Chilean matorral, and allows to better frame this region in the context of the different evolutionary histories among MTE.

## Supporting information

S1 Table List of species, localities of seed sampling, date of seed collection and experiments, seed storage time, number of seeds per Petri dish (block) and number of blocks used in the experiments. NP = National Park; NR = Natural Reserve; NrP = Natural Park; SN = Sanctuary of Nature. (E) = Endemic to Chile. Nomenclature follows Zuloaga et al. [42].
(DOCX)

S2 Table Statistical results of the Glz analyses evaluating the effect of seed storage time on the seed responses to the heat shock treatments across species. Significant P values are highlighted in bold. Wald-z tests were performed in the cases of binomial family and Wald-t test in the cases of quasibinomial family. Probability values (0,1) of stimulation and inhibition of each species were extracted from Table 2 (see S1 File).
(DOCX)

S3 Table Statistical results of the GLMM analyses evaluating the effect of heat-shock (100°C and 120°C, 5 min) on the probability of seed germination and survival of 21 common woody species from the Chilean matorral. Significant P values are highlighted in bold. (E) = endemic to Chile. NC = Not converged model due to zero germination only under heat shock treatment (a negative effect was assumed). NG = Not analyzed due to zero germination in all treatments.NA = Not addressed. Nomenclature follows Zuloaga et al. [42].
(DOCX)

S4 Table Additional information on matorral woody species for which the effect of heat or smoke on seed germination has been addressed in previous studies. (E) = endemic to Chile; (H) = Heat shock treatment; (S) = Smoke treatment; (+) Positive response; (-) Negative response; (0) No response; (NG) = No germination. See references in the main text. Nomenclature follows Zuloaga et al. [42].
(DOCX)

S1 Supporting Information Experiment on *Cryptocarya alba* seeds. (DOCX)

S1 Fig Example of differential fungal infection of *Peumus boldus* seeds in control and heated samples. (TIF)

S1 File Excel file with the raw data. (XLSX)
