# Supplementary material for: Postfire responses of the woody flora of Central Chile: Insights from a germination experiment
Source: PLoS One. 2017 Jul 12;12(7):e0180661. doi: 10.1371/journal.pone.0180661 (PMC5507535; doi:10.1371/journal.pone.0180661)
Supplement: S1 Table — NP = National Park; NR = Natural Reserve; NrP = Natural Park; SN = Sanctuary of Nature. (E) = Endemic to Chile. Nomenclature follows Zuloaga et al. [42]. (DOCX) [file pone.0180661.s001.docx]

**SUPPORTING INFORMATION**

**S1 Table: List of species, localities of seed sampling, date of seed collection and experiments, seed storage time, number of seeds per Petri dish (block) and number of blocks used in the experiments.**

| **Species** | **Locality (Region)** | **Collection date**  **(mm/yy)** | **Experiment date (mm/yy)** | **Storage time**  **(months)** | **Seeds per block** | **Number of blocks** |
| --- | --- | --- | --- | --- | --- | --- |
| *Acacia caven* | Río Clarillo NR (Metropolitan) | 03/13 | 01/14 | 10 | 45 | 4 |
| *Azara petiolaris* (E) | La Campana NP (Valparaíso) | 05/13 | 09/13 | 4 | 50 | 4 |
| *Baccharis linearis* | Quebrada de La Plata (Metropolitan) | 03/08 | 01/09 | 10 | 30 | 6 |
| *Budleja globosa* | Río Clarillo NR (Metropolitan) | 03/13 | 09/13 | 6 | 40 | 4 |
| *Cestrum parqui* | Río Clarillo NR (Metropolitan) | 03/13 | 09/13 | 6 | 50 | 4 |
| *Colliguaja integerrima* | Río Clarillo NR (Metropolitan) | 03/13 | 09/13 | 6 | 25 | 4 |
| *Colliguaja odorifera* (E) | Río Clarillo NR (Metropolitan); Cuesta La Dormida (Metropolitan); Aguas de Ramón NrP (Metropolitan) | 01/13 | 01/14 | 12 | 40 | 4 |
| *Cryptocarya alba* (E) | Florida (Bío-Bío) | 04/14 | 10/14 | 6 | 27 | 4 |
| *Kageneckia angustifolia* (E) | Yerba Loca SN (Metropolitan) | 03/13 | 01/14 | 10 | 50 | 4 |
| *Kageneckia oblonga* (E) | Aguas de Ramón NrP (Metropolitan) | 03/13 | 01/14 | 10 | 50 | 4 |
| *Lithraea caustica* (E) | Colliguay ( Valparaíso ) | 05/08 | 01/09 | 8 | 30 | 6 |
| *Maytenus boaria* | Río Clarillo NR (Metropolitan) | 03/13 | 01/14 | 10 | 40 | 4 |
| *Muehlenbeckia hastulata* | Quintero (V Región) | 05/08 | 01/09 | 8 | 30 | 6 |
| *Peumus boldus* (E) | Colliguay (Valparaíso) | 03/13 | 01/14 | 10 | 50 | 4 |
| *Podanthus mitiqui* (E) | Río Clarillo NR (Metropolitan) | 05/13 | 09/13 | 4 | 50 | 4 |
| *Otholobium glandulosum* (E) | Río Clarillo NR (Metropolitan) | 03/13 | 10/14 | 19 | 50 | 4 |
| *Quillaja saponaria* (E) | Río Clarillo NR (Metropolitan) | 03/13 | 10/14 | 10 | 50 | 4 |
| *Retanilla ephedra* (E) | Río Clarillo NR (Metropolitan) | 05/13 | 10/14 | 8 | 20 | 4 |
| *Retanilla trinervia* (E) | Río Clarillo NR (Metropolitan) | 05/13 | 10/14 | 8 | 50 | 4 |
| *Senna candoliana* (E) | La Campana NP (Valparaíso) | 05/13 | 10/14 | 17 | 50 | 4 |
| *Sophora macrocarpa* (E) | Rodelillo (Valparaíso) | 05/13 | 10/14 | 17 | 30 | 4 |

NP = National Park; NR = Natural Reserve; NrP = Natural Park; SN = Sanctuary of Nature. (E) = Endemic to Chile. Nomenclature follows Zuloaga *et al.* [42].
