# Supplementary material for: Postfire responses of the woody flora of Central Chile: Insights from a germination experiment
Source: PLoS One. 2017 Jul 12;12(7):e0180661. doi: 10.1371/journal.pone.0180661 (PMC5507535; doi:10.1371/journal.pone.0180661)
Supplement: S2 Table — Significant P values are highlighted in bold. Wald-z tests were performed in the cases of binomial family and Wald-t test in the cases of quasibinomial family. Probability values (0,1) of stimulation and inhibition of each species were extracted from Table 2 (see S1 File). (DOCX) [file pone.0180661.s002.docx]

**SUPPORTING INFORMATION**

**S2 Table: Statistical results of the Glz analyses evaluating the effect of seed storage time on the seed responses to the heat shock treatments across species.**

| **Variables** | **Germination** | | | **Survival** | | |
| --- | --- | --- | --- | --- | --- | --- |
|  | ***β *(SE)** | ***z/t*** | ***P*** | ***β *(SE)** | ***z/t*** | ***P*** |
| *Probability of stimulation* |  |  |  |  |  |  |
| 100°C | 0.26 (0.14) | 1.77 | 0.080 | 0.34 (0.18) | 1.91 | 0.056 |
| 120°C | 0.16 (0.16) | 0.97 | 0.331 | 0.22 (1.17) | 1.26 | 0.207 |
| *Probability of inhibition* |  |  |  |  |  |  |
| 100°C | -10.75 (1822.51) | -0.01 | 0.995 | -9.76 (1318.68) | -0.01 | 0.994 |
| 120°C | -0.11 (0.12) | -0.97 | 0.333 | -0.07 (0.11) | -0.60 | 0.545 |

Significant P values are highlighted in bold. Wald-z tests were performed in the cases of binomial family and Wald-t test in the cases of quasibinomial family. Probability values (0,1) of stimulation and inhibition of each species were extracted from Table 2 (see raw data in S1 file).
