# Supplementary material for: Postfire responses of the woody flora of Central Chile: Insights from a germination experiment
Source: PLoS One. 2017 Jul 12;12(7):e0180661. doi: 10.1371/journal.pone.0180661 (PMC5507535; doi:10.1371/journal.pone.0180661)
Supplement: S3 Table — Significant P values are highlighted in bold. (E) = endemic to Chile. NC = Not converged model due to zero germination only under heat shock treatment (a negative effect was assumed). NG = Not analyzed due to zero germination in all treatments.NA = Not addressed. Nomenclature follows Zuloaga et al. [42]. (DOCX) [file pone.0180661.s003.docx]

**SUPPORTING INFORMATION**

**S3 Table:** **Statistical results of the GLMM analyses evaluating the effect of heat-shock (100°C and 120°C, 5 min) on the probability of seed germination and survival of 21 common woody species from the Chilean matorral.**

| **Species** | **Germination** | | | **Survival** | | |
| --- | --- | --- | --- | --- | --- | --- |
|  | ***β *(SE)** | ***z*** | ***P*** | ***β *(SE)** | ***z*** | ***P*** |
| *Acacia caven* |  |  |  |  |  |  |
| Intercept | -3.0 (0.5) | -6.0 | **<0.001** | 3.3 (0.7) | 4.5 | **<0.001** |
| 100°C | -0.5 (0.7) | -0.7 | 0.484 | 0.5 (1.1) | 0.5 | 0.646 |
| 120°C | -1.0 (0.8) | -1.3 | 0.183 | 1.0 (1.1) | 0.9 | 0.392 |
| *Azara petiolaris* (E) |  |  |  |  |  |  |
| Intercept | NC | NC | NC | NC | NC | NC |
| 100°C | NC | NC | NC | NC | NC | NC |
| 120°C | NC | NC | NC | NC | NC | NC |
| *Baccharis linearis* |  |  |  |  |  |  |
| Intercept | 1.5 (0.4) | 3.8 | **<0.001** | NA | NA | NA |
| 100°C | 0.0 (0.5) | 0.0 | 0.978 | NA | NA | NA |
| 120°C | NA | NA | NA | NA | NA | NA |
| *Buddleja globosa* |  |  |  |  |  |  |
| Intercept | -2.4 (0.4) | -5.6 | **<0.001** | -1.8 (0.2) | -8.0 | **<0.001** |
| 100°C | -1.5 (0.7) | -2.1 | **0.039** | -2.1 (0.6) | -3.4 | **<0.001** |
| 120°C | NC | NC | NC | NC | NC | NC |
| *Cestrum parqui* |  |  |  |  |  |  |
| Intercept | NC | NC | NC | 4.5 (0.8) | 5.5 | **<0.001** |
| 100°C | NC | NC | NC | -7.0 (1.0) | -6.9 | **<0.001** |
| 120°C | NC | NC | NC | -6.7 (1.0) | -6.7 | **<0.001** |
| *Colliguaja integerrima* |  |  |  |  |  |  |
| Intercept | 0.6 (0.4) | 1.7 | 0.080 | 1.2 (0.4) | 2.7 | **0.007** |
| 100°C | -2.6 (0.6) | -4.4 | **<0.001** | -0.5 (0.6) | -0.7 | 0.448 |
| 120°C | NC | NC | NC | -0.1 (0.6) | -0.1 | 0.914 |
| *Colliguaja odorifera* (E) |  |  |  |  |  |  |
| Intercept | -0.8 (0.2) | -3.7 | **<0.001** | -0.7 (0.2) | 3.4 | **<0.001** |
| 100°C | 0.3 (0.3) | 1.0 | 0.326 | 0.3 (0.3) | 0.9 | 0.379 |
| 120°C | -2.3 (0.4) | -5.1 | **<0.001** | -0.7 (0.3) | -2.2 | **0.027** |
| *Cryptocarya alba* (E) |  |  |  |  |  |  |
| Intercept | 1.5 (0.2) | 6.0 | **<0.001** | 1.5 (0.2) | 6.0 | **<0.001** |
| 100°C | -0.3 (0.3) | -0.8 | 0.393 | -0.3 (0.3) | -0.9 | 0.319 |
| 120°C | -0.1(0.3) | -0.3 | 0.782 | -0.1(0.3) | -0.4 | 0.782 |
| *Kageneckia angustifolia* (E) |  |  |  |  |  |  |
| Intercept | 2.3 (0.5) | 4.8 | **<0.001** | 2.3 (0.4) | 5.8 | **<0.001** |
| 100°C | 0.2 (0.7) | 0.4 | 0.674 | 0.2 (0.6) | 0.4 | 0.710 |
| 120°C | -5.3 (0.8) | -7.0 | **<0.001** | -4.4 (0.6) | -7.7 | **<0.001** |
| *Kageneckia oblonga* (E) |  |  |  |  |  |  |
| Intercept | 3.1 (0.4) | 7.5 | **<0.001** | 3.1 (0.4) | 7.3 | **<0.001** |
| 100°C | -0.1 (0.6) | -0.2 | 0.875 | 0.0 (0.6) | 0.1 | 0.953 |
| 120°C | -3.5 (0.5) | -7.0 | **<0.001** | -3.0 (0.5) | -5.8 | **<0.001** |
| *Lithraea caustica* (E) |  |  |  |  |  |  |
| Intercept | -3.2 (0.4) | -7.4 | **<0.001** | NA | NA | NA |
| 100°C | 2.0 (0.5) | 4.1 | **<0.001** | NA | NA | NA |
| 120°C | NA | NA | NA | NA | NA | NA |
| *Maytenus boaria* |  |  |  |  |  |  |
| Intercept | -3.6 (1.2) | -3.1 | **0.002** | 0.7 (0.5) | 1.5 | 0.131 |
| 100°C | -1.8 (1.6) | -1.1 | 0.260 | -1.0 (0.7) | -1.4 | 0.146 |
| 120°C | NC | NC | NC | NC | NC | NC |
| *Muehlenbekia hastulata* |  |  |  |  |  |  |
| Intercept | -5.2 (1-0) | -5.2 | **<0.001** | NA | NA | NA |
| 100°C | 2.3 (1.0) | 2.2 | **0.025** | NA | NA | NA |
| 120°C | NA | NA | NA | NA | NA | NA |
| *Peumus boldus* (E) |  |  |  |  |  |  |
| Intercept | NG | NG | NG | 0.0 (0.0) | 0.0 | 1.0 |
| 100°C | NG | NG | NG | 1.0 (0.4) | 2.4 | **0.014** |
| 120°C | NG | NG | NG | 2.6 (0.5) | 5.5 | **<0.001** |
| *Podanthus mitiqui* (E) |  |  |  |  |  |  |
| Intercept | NC | NC | NC | 0.5 (1.0) | 0.5 | 0.628 |
| 100°C | NC | NC | NC | -3.8 (1.5) | -2.5 | **0.013** |
| 120°C | NC | NC | NC | -5.2 (1.8) | -2.9 | **0.003** |
| *Otholobium glandulosum* (E) |  |  |  |  |  |  |
| Intercept | 1.9 (0.2) | 9.1 | **<0.001** | 5.3 (1.0) | 5.2 | **<0.001** |
| 100°C | 0.8 (0.4) | 2.2 | **0.028** | -1.1 (1.1) | -0.9 | 0.349 |
| 120°C | -3.7 (0.3) | -12.6 | **<0.001** | -7.1 (1.0) | -6.7 | **<0.001** |
| *Quillaja saponaria* (E) |  |  |  |  |  |  |
| Intercept | -1.7 (0.3) | -5.8 | **<0.001** | -0.2 (0.4) | -5.4 | 0.586 |
| 100°C | -0.4 (0.4) | -0.9 | 0.347 | 0.2 (0.5) | 0.4 | 0.666 |
| 120°C | NC | NC | NC | 0.8 (0.5) | 1.5 | 0.137 |
| *Retanilla ephedra* (E) |  |  |  |  |  |  |
| Intercept | -3.2 (0.7) | -4.6 | **<0.001** | 1.2 (0.3) | 4.3 | **<0.001** |
| 100°C | 0.5 (0.9) | 0.5 | 0.597 | 0.1 (0.4) | 0.4 | 0.685 |
| 120°C | 0.9 (0.9) | 1.0 | 0.297 | 0.3 (0.4) | 0.8 | 0.429 |
| *Retanilla trinervia* (E) |  |  |  |  |  |  |
| Intercept | -3.2 (0.4) | -8.8 | **<0.001** | 1.2 (0.4) | 3.1 | **0.002** |
| 100°C | 0.6 (0.4) | 1.3 | 0.194 | -0.5 (0.5) | -1.0 | 0.333 |
| 120°C | 1.8 (0.4) | 4.5 | **<0.001** | -0.1 (0.5) | -0.2 | 0.846 |
| *Senna candolleana* (E) |  |  |  |  |  |  |
| Intercept | -3.3 (0.4) | -8.6 | **<0.001** | 0.8 (0.1) | 5.2 | **<0.001** |
| 100°C | 1.0 (0.4) | 2.2 | **0.028** | 3.1 (0.5) | 5.8 | **<0.001** |
| 120°C | -0.6 (0.6) | -0.9 | 0.365 | 3.8 (0.7) | 5.2 | **<0.001** |
| *Sophora macrocarpa* (E) |  |  |  |  |  |  |
| Intercept | -1.6 (0.3) | -5.0 | **<0.001** | 0.9 (0.5) | 2.0 | **0.042** |
| 100°C | 0.3 (0.4) | 0.7 | 0.478 | 3.0 (0.9) | 3.4 | **<0.001** |
| 120°C | 1.0 (0.4) | 2.3 | **0.019** | 1.0 (0.7) | 1.5 | 0.132 |

Significant P values are highlighted in bold. (E) = endemic to Chile. NC = Not converged model due to zero germination just under heat shock treatment, and thus, a negative effect was assumed. NG = Not analyzed due to zero germination in all treatments. NA = Not addressed. Nomenclature follows Zuloaga et al. [42].
