# Supplementary material for: Postfire responses of the woody flora of Central Chile: Insights from a germination experiment
Source: PLoS One. 2017 Jul 12;12(7):e0180661. doi: 10.1371/journal.pone.0180661 (PMC5507535; doi:10.1371/journal.pone.0180661)
Supplement: S4 Table — (E) = endemic to Chile; (H) = Heat shock treatment; (S) = Smoke treatment; (+) Positive response; (-) Negative response; (0) No response; (NG) = No germination. See references in the main text. Nomenclature follows Zuloaga et al. [42]. (DOCX) [file pone.0180661.s004.docx]

**SUPPORTING INFORMATION**

**S4 Table:** **Additional information on matorral woody species for which the effect of heat or smoke on seed germination has been addressed in previous studies.**

| **Species** | **Family** | **Growth form** | **Response to fire cues** |
| --- | --- | --- | --- |
| *Baccharis vernalis* (E) | Asteraceae | Shrub | (S)+ [35] |
| *Colletia hystrix* | Rhamnaceae | Shrub | (H)NG [34]; (S)NG [35] |
| *Eccremocarpus scaber* | Bignoniaceae | Woody vine | (S)- [35] |
| *Escallonia pulverulenta* (E) | Escalloniaceae | Shrub | (S)0 [35] |
| *Happlopapus schumanii* (E) | Asteraceae | Shrub | (S)- [35] |
| *Schinus polygamus* (E) | Anacardiaceae | Tree | (H)0 [34] |
| *Solanum crispum* | Solanaceae | Shrub | (S)NG [35] |
| *Trevoa quinquenervia* (E) | Rhamnaceae | Shrub | (S)+ [35] |

(E) = endemic to Chile; (H) = Heat shock treatment; (S) = Smoke treatment; (+) Positive response; (-) Negative response; (0) No response; (NG) = No germination. See references in the main text. Nomenclature follows Zuloaga et al. [42].
