# Supplementary material for: Postfire responses of the woody flora of Central Chile: Insights from a germination experiment
Source: PLoS One. 2017 Jul 12;12(7):e0180661. doi: 10.1371/journal.pone.0180661 (PMC5507535; doi:10.1371/journal.pone.0180661)
Supplement: S1 Supporting Information — (DOCX) [file pone.0180661.s005.docx]

**SUPPORTING INFORMATION**

**S1 Supporting information: Experiment on *Cryptocarya alba* seeds**

In the case of *C. alba*, the pericarp of the seeds was manually removed before the experiments since it is known that it has inhibitory effects on seed germination [49]. To explore whether heat-shock might break a possible seed dormancy imposed by the pericarp in *C. alba*, we performed a previous experiment (the same as explained above) without removing the pericarp and we found no germination in any treatment. We observed that all seeds were dark pink dyed by the lixiviated of the pericarp. This lixiviated stained the seed with the same color (dark pink) of the TTC solution, hampering the interpretation of the viability test. However, all seeds seemed to be healthy, and thus we assumed that the pericarp lixiviates inhibited the germination without killing the seeds [49].

See references in the main text.
