# Supplementary figures and images for: Postfire responses of the woody flora of Central Chile: Insights from a germination experiment
Source: PLoS One. 2017 Jul 12;12(7):e0180661. doi: 10.1371/journal.pone.0180661 (PMC5507535; doi:10.1371/journal.pone.0180661)

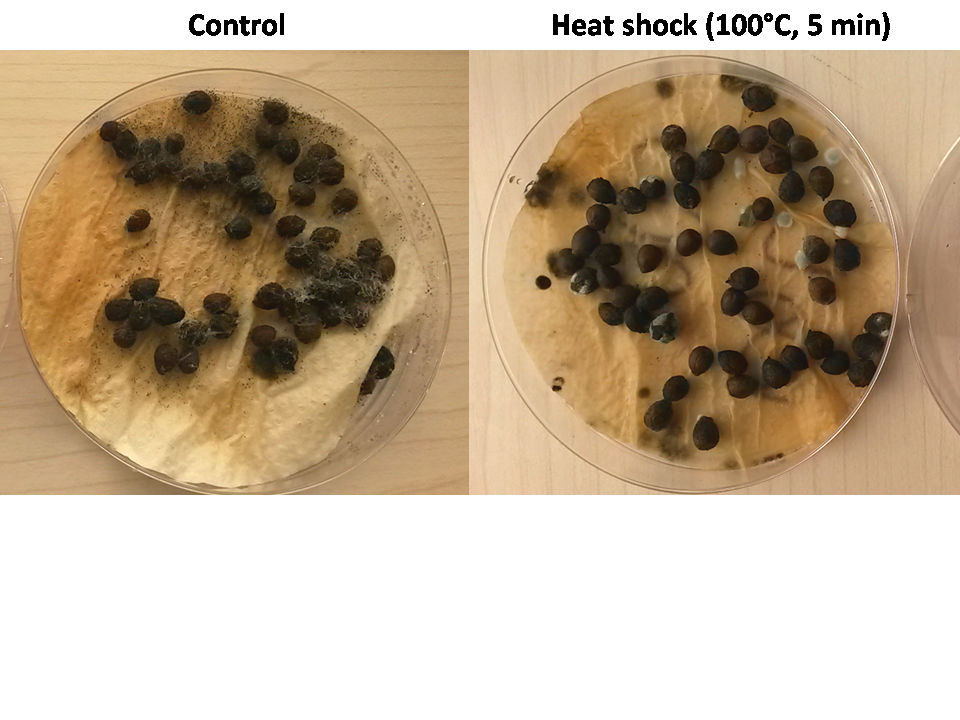

Supplement: S1 Fig — (TIF) [file pone.0180661.s006.tif]
